# Acetylcholine and noradrenaline differentially regulate hippocampus-dependent spatial learning and memory

**DOI:** 10.1093/braincomms/fcac338

**Published:** 2022-12-22

**Authors:** Gioacchino de Leo, Rosario Gulino, Marino Coradazzi, Giampiero Leanza

**Affiliations:** Neurogenesis and Repair Lab., B.R.A.I.N. Centre for Neuroscience, Department of Life Sciences, University of Trieste, Via Fleming 2, 34127 Trieste, Italy; Neurophysiology Lab., Department of Biomedical and Biotechnological Sciences, University of Catania, Via S. Sofia 89, 95123 Catania, Italy; Molecular Preclinical and Translational Imaging Research Centre—IMPRonTE, University of Catania, Catania, Italy; Neurogenesis and Repair Lab., B.R.A.I.N. Centre for Neuroscience, Department of Life Sciences, University of Trieste, Via Fleming 2, 34127 Trieste, Italy; Neurogenesis and Repair Lab., Department of Drug and Health Sciences, University of Catania, Via S. Sofia 64, 95125 Catania, Italy; Molecular Preclinical and Translational Imaging Research Centre—IMPRonTE, University of Catania, Catania, Italy

**Keywords:** acetylcholine, noradrenaline, immunotoxin, reference and working memory, sympathetic sprouting

## Abstract

Severe loss of cholinergic neurons in the basal forebrain nuclei and of noradrenergic neurons in the locus coeruleus are almost invariant histopathological hallmarks of Alzheimer’s disease. However, the role of these transmitter systems in the spectrum of cognitive dysfunctions typical of the disease is still unclear, nor is it yet fully known whether do these systems interact and how. Selective ablation of either neuronal population, or both of them combined, were produced in developing animals to investigate their respective and/or concurrent contribution to spatial learning and memory, known to be severely affected in Alzheimer’s disease. Single or double lesions were created in 4–8 days old rats by bilateral intraventricular infusion of two selective immunotoxins. At about 16 weeks of age, the animals underwent behavioural tests specifically designed to evaluate reference and working memory abilities, and their brains were later processed for quantitative morphological analyses. Animals with lesion to either system alone showed no significant reference memory deficits which, by contrast, were evident in the double-lesioned subjects. These animals could not adopt an efficient search strategy on a given testing day and were unable to transfer all relevant information to the next day, suggesting deficits in acquisition, storage and/or recall. Only animals with single noradrenergic or double lesions exhibited impaired working memory. Interestingly, ablation of cholinergic afferents to the hippocampus stimulated a robust ingrowth of thick fibres from the superior cervical ganglion which, however, did not appear to have contributed to the observed cognitive performance. Ascending cholinergic and noradrenergic afferents to the hippocampus and neocortex appear to be primarily involved in the regulation of different cognitive domains, but they may functionally interact, mainly at hippocampal level, for sustaining normal learning and memory. Moreover, these transmitter systems are likely to compensate for each other, but apparently not via ingrowing sympathetic fibres.

## Teaser

Ascending cholinergic and noradrenergic inputs regulate cognitive abilities and their dysfunction is a major hallmark in pathologies with memory loss. Here, de Leo *et al*. report that these inputs converge to hippocampus to regulate different cognitive domains but they functionally interact and can efficiently compensate for each other when disrupted.

## Graphical Abstract

**Graphical Abstract:**
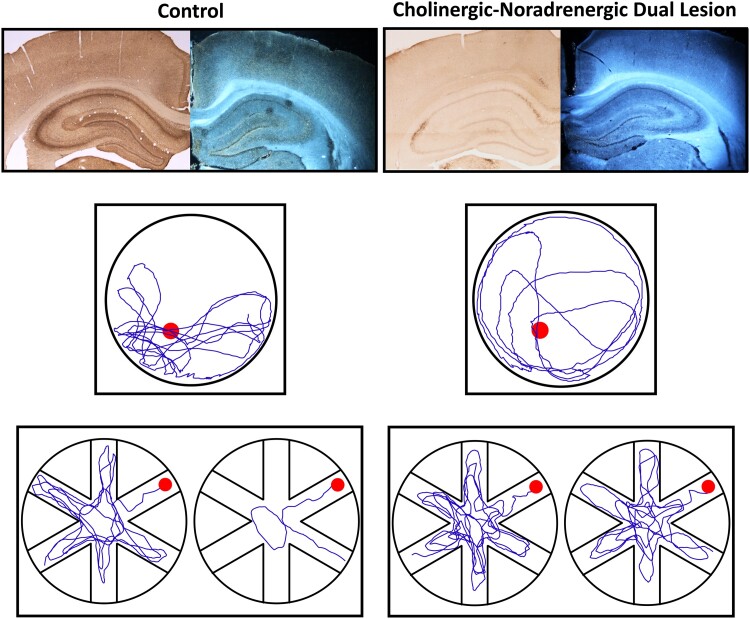

## Introduction

Alzheimer’s disease (AD) is the most common cause of dementia in the elderly populations and is pathologically defined by a dramatic loss of cholinergic neurons in the basal forebrain (BF) as well as by regional accumulation of misfolded beta amyloid peptides (Aß) in extracellular plaques and of phosphorilated tau protein in intraneuronal neurofibrillary tangles (NFTs).^1-3^ In spite of the popularity acquired by the so-called cholinergic hypothesis of AD in the past decades,^4-7^ it has become increasingly clear that the complex clinical manifestations of the disease cannot be related to cholinergic atrophy alone.^8,9^ In fact, other systems are likely involved and, among them, noradrenaline (NA)-rich neurons in the locus coeruleus (LC) have long been suggested to play a role.^10-13^ Alterations in the LC NA system are among the earliest signs of AD-type pathology, being detected even decades prior to the appearance of clinical symptoms,^14,15^ and correlate highly to both neurofibrillary pathology and the severity of cognitive impairments, when overtly present.^16,17^

However, although the extent of noradrenergic cell depletion in the LC has been shown to exceed that of cholinergic neurons in the BF,^18^ its importance as a potential target for therapeutic interventions in AD has been underestimated so far. In fact, only in relatively recent years has degeneration of LC neurons and the associated NFT pathology been widely recognized as a prominent AD feature.^19-26^

Notwithstanding, the exact noradrenergic contribution to the cognitive impairments and pathogenesis that characterize AD is still poorly understood. More importantly, while integrity of both the BF cholinergic and the LC noradrenergic transmitter systems appears to be a prerequisite for normal cognitive abilities, it is still unclear if these two systems cooperate to maintain these abilities in a fully operational state or whether are they functionally segregated, each participating to the regulation of a specific aspect of cognitive performance.

The issue of a possible functional interaction between the BF cholinergic and the LC noradrenergic projection systems in the regulation of cognitive abilities is not a novel one.^27^ In some studies, the interaction was reported as synergistic, with NA depletion enhancing the spatial learning impairments induced by cholinergic blockade or lesioning,^28-30^ whereas either cholinergic or noradrenergic blockade alone produced no clear-cut effects on a maze-based spatial memory task.^31^ Other studies, by contrast, have proposed a different picture, suggesting that such interaction may be either antagonistic^32-34^ or even absent/very mild at best.^35^ It should be noted, however, that none of the blocking/lesioning approaches used in these studies has proven to be selective for cholinergic and/or noradrenergic neurons, which has long hampered the understanding of the functional consequences of their individual or simultaneous loss. The availability of highly selective immunotoxins, based on the coupling of ribosome-inactivating proteins with cell-specific ligands, such as antibodies, has offered interesting alternatives not previously available to address these issues,^36^ including the possibility to refine the analysis and interpretation of the observed cognitive outcomes, but a cholinergic–noradrenergic dual immunolesioning approach has never be tempted so far.

In the present study, selective ablation of the BF cholinergic or the LC noradrenergic neurons, or both these neuronal populations combined, were carried out in developing animals, which then underwent extensive behavioural analyses during adulthood. The reason for producing neonatal, as opposed to adult, immunolesions is related to the possibility to follow the development and consolidation of the lesion-perturbed cognitive abilities over time, and to address possible additive/synergistic effects. Moreover, this early lesion, at least as far as both cholinergic and noradrenergic transmitter systems are concerned, has been observed to result in no detectable loss of cerebellar Purkinje cells,^37,38^ considered to be a confounding factor when interpreting its functional outcomes.^39,40^ The aim of the studies, therefore, was to investigate whether double-lesioned animals would exhibit cognitive impairments of different kinds and magnitudes, compared with those possibly detected in subjects with either lesion alone. Moreover, the issue of a possible cholinergic–noradrenergic interplay in the regulation of cognitive abilities was also addressed.

## Materials and methods

### Subjects and experimental design

A total of 60 equally distributed male and female Wistar rats (provided by the animal facility at the University of Trieste) from 6 different litters were used. The pups were randomly allocated into groups subjected to either of four different treatments: (i) bilateral intraventricular injection of 192 IgG-saporin (ACh lesion, *n* = 15); (ii) bilateral intraventricular injection of anti-DBH-saporin (NA lesion, *n* = 15); (iii) bilateral intraventricular injection of both 192 IgG-saporin and anti-DBH-saporin (ACh/NA double lesion, *n* = 15) and (iv) bilateral intraventricular injection of vehicle solution (Vehicle, *n* = 8). The remaining pups were not injected and served as unoperated controls (Intact, *n* = 7). Sample size was estimated by power analysis, as reported by Cohen.^41^ Litters (one per cage) were fostered by the mothers until weaning at 21 days of age. The rats were housed in high efficiency, particulate air-filtered double decker cage units (Tecniplast, Italy) under standard conditions of light, temperature and humidity with *ad libitum* access to food and water.

The animals’ general status (increase in body weight, presence of normal sensory and motor skills) was assessed at about 4 weeks post-surgery (i.e. about 5 weeks of age), and then every 2 weeks up to about 16 weeks post-lesion, when the animals underwent the sequential administration of behavioural tests designed to evaluate reference and working memory abilities. Upon completion of the last testing session, at about 20 weeks post-lesion, the animals were perfused and the brains processed for histo- and immunohistochemistry (see Supplementary material).

Animal care and handling followed the Italian Guidelines for Animal Care (26/2014), which are in compliance with the European Community Council Directives (2010/63/EU) and were approved by the Ethical Committee at the University of Trieste (ref. no. 1313LEA12).

### Lesion surgery

Selective lesions of the developing cholinergic and/or noradrenergic systems were performed on 4 and 8-day-old (post-natal day, PD, 4 and 8) pups under hypothermic anaesthesia.^37,38^ The 192 IgG-saporin or the anti-DBH-saporin immunotoxins (Advanced Targeting Systems, San Diego, CA, USA) were injected at a dose of 0.4 µg using 10 µl microsyringes in a volume of 5 µl/side of vehicle solution (sterile phosphate-buffered saline, PBS; each injection thus contained half of the total dose) into the lateral ventricles at the following coordinates (in mm, relative to bregma and outer skull surface): AP = −0.6, L = ±0.8 V = −2.1.

Lesioning of either BF cholinergic or LC noradrenergic neurons alone was carried out using a counterbalanced injection design, with half of the animals in the groups receiving the 192 IgG-saporin (or the anti-DBH-saporin) immunotoxin at PD4 and the remaining animals at PD8. For the double lesions a counterbalanced, two-stage administration procedure was adopted, with half of the animals in the group receiving one toxin at PD4 and the other toxin at PD8, such order being switched in the remaining animals. In pilot experiments, the simultaneous administration of both toxins in the same bolus was observed to reduce their respective lesioning efficacy by 10–20%; therefore, this two-stage procedure was consistently adopted here. Substances were injected at a speed of 2 µl/min, allowing 3 min for diffusion before the cannula was retracted. As to sham lesions, sterile PBS was injected using the same coordinates, volume and speed. Local treatment with 2.5% lidocaine-prilocaine cream (EMLA, AstraZeneca, Italy) was consistently carried out so as to minimize pain and discomfort during and after the surgical procedures. After each surgery, the pups were allowed to fully recover and reacquire normal body temperature under a filament bulb, prior to being returned to the mothers and left undisturbed.

### Behavioural analyses

#### Motor tests

All testing was consistently carried out between 9:00 am and 3:00 pm. In order to assess unspecific motor disturbances possibly induced by the toxin treatments,^42,43^ simple motor tests of limb strength and coordination were administered every second week to all animals starting from about 4 weeks post-lesion.^38^ Briefly, locomotive form and support were assessed after placing the rat onto a wooden ramp (80 cm long, 4 cm wide), which was connected to the animal’s home cage and was maintained either horizontal or inclined at a 45° angle. An inclined (75°) 80 × 30 cm framed grid made of coarse-mesh chicken wire was also used, where the rats were placed head-down, being requested to reverse the direction and climb onto it.

#### Morris water maze

Spatial learning and memory abilities were evaluated using the Morris water maze (MWM) task.^44^ The apparatus consisted in a circular pool, 150 cm in diameter and 50 cm deep filled to a depth of 35 cm with room temperature (20°C) water. Four equally spaced points, conventionally indicated as North, South, East and West, served as start locations, and divided the tank into four quadrants. The tank was located in a corner of a well-lit rectangular (3.5 × 4.50 m) room containing many external cues (i.e. brightly coloured 50 × 70 cm posters on the walls, at least two on each wall) that could be used by the animals for orientation. A circular platform (10 cm diameter) was anchored to the bottom of the pool in one of the quadrants, with its top 2 cm below (and thus invisible from) the water surface, onto which the animal could climb to escape. Four annuli were defined as a circular area in the middle of each quadrant, corresponding to the site where the platform would have been, if placed in that quadrant.

Starting from about 16 weeks post-lesion, the rats were tested in the MWM using a 4 trials a day schedule, with a 30 s inter-trial interval. After receiving a free 60-s swim to become familiar with the swimming pool environment, all animals underwent a 3-days cued learning session, during which the platform was moved to a new location on each trial, and its position was made visible by a 10 × 15 cm striped flag. This test was designed to evaluate the occurrence of non-cognitive (e.g. visual) impairments, possibly induced by the lesions.

Three days after the conclusion of the cued test, and for seven consecutive days, a place learning task was administered, during which the hidden platform remained in the same fixed position (typically the southwest, SW, quadrant). On each trial, the rat was placed into the water facing the wall of the tank at one of the starting positions, and given 60 s to find the platform and climb onto it. Once the rat had reached the platform, it was allowed to rest for the subsequent 30 s, before being picked up and placed in the next predetermined position. Animals that failed to locate the platform within 60 s were gently guided to, and allowed to rest onto it for 30 s. The latency to find the platform, the distance swum and the swim speed were recorded by a computer-based video tracking system. On the final day of testing (day 7), after the last trial, the platform was removed and a fifth spatial probe trial was administered, in which the rat was allowed to swim freely for 60 s. The swim path was plotted, and the distance swum and the number of annulus crossings in each quadrant were recorded.

In order to detect possible differences in the search strategy adopted during the execution of the reference memory test, a blinded observer evaluated the swim paths from the subjects in the various treatment groups. In particular, three main patterns of search behaviour were considered for the analysis:^45^ (1) use of a spatial search strategy. This pattern occurred when the animals efficiently used the extramaze cues to locate the hidden platform, and was characterized by frequent and sharp changes of swim direction; (2) use of a non-spatial search strategy. This pattern occurred when the animals used mainly intramaze (i.e. egocentric), instead of extramaze (i.e. allocentric) cues, with a complex, poorly focused, circular swimming that may nevertheless result in platform location; (3) no search strategy used. This pattern occurred when the animals did not exhibit any obvious search behaviour, and was characterized by the frequent adoption of a thigmotaxic swimming, very close to, or in contact with the wall of the pool.

#### Radial arm water maze

The radial arm water maze (RAWM) task^46,47^ was used to evaluate spatial working memory abilities. Briefly, twelve plexiglas walls (50 cm length × 50 cm height) were positioned within the circular pool, so as to create six radially distributed swimming arms and a central open area. The platform was placed at the end of one arm and its position was changed on each of five consecutive testing days, but it was kept constant over the five trials of a given day. On each trial, the animal was released from a different start arm and given 60 s to locate the platform, with a 30-s inter-trial time. Entering an incorrect arm (i.e. that did not host the platform) or an already visited arm was counted as an entry error. For each trial, the latency to reach the platform and the number of arm selection errors were recorded. Moreover, the difference in latency or error scores between trials 1 and 2, calculated as a percentage of trial 1, was adopted as a further estimate of animals’ performance (savings).

### Statistical analysis

Statistical comparisons were carried out using one-way ANOVA, two-way mixed ANOVA, three-way ANOVA or Pearson chi-square test, where appropriate. In cases in which the overall ANOVA indicated significant effects, *post hoc* analyses were conducted using the Tukey HSD test. Data are presented as either means ± standard error of the mean (SEM) or % pattern categories (for the analysis of swim strategy), and differences were considered significant at *P* < 0.05.

### Data availability

The data that support the findings of this study are available from the corresponding author, upon reasonable request.

## Results

### General observations

In no case did the toxins, injected either alone or in combination, induce any mortalities, seizures or unspecific tissue disruption that would have interfered with the analyses, and no differences in the efficiency of the lesion was detected as a result of the counterbalanced, or two-stage, injection design adopted. Thus, all animals, irrespective of their treatment, increased in body weight, and exhibited fairly normal sensory-motor functioning when evaluated in both the bridge and grid tests at about 5 weeks of age (Table 1). Sham-lesioned animals never differed from unoperated controls on any of the functional or morphometric parameters analysed (0.1618 ≤ *P* ≤ 0.966). These subgroups were therefore combined into a single control group for statistical analyses and illustrations.

**Table 1:** Motor performance

Group	Equilibrium time on ramp (%)	Latency to cross ramp (s)	Latency to reverse on grids (s)	Number of falls in grids
Intact (7)	98.1 ± 9.9	5.9 ± 0.4	5.1 ± 0.9	2.1 ± 0.4
Vehicle (8)	95.8 ± 12.6	7.1 ± 0.5	5.4 ± 0.5	2.0 ± 0.6
ACh lesion (15)	96.6 ± 9.1	7.0 ± 0.3	5.3 ± 0.5	2.5 ± 0.5
NA lesion (15)	97.4 ± 5.1	6.6 ± 0.3	5.5 ± 0.6	2.6 ± 0.5
ACh–NA lesion (15)	97.0 ± 7.8	6.5 ± 0.3	5.6 ± 0.6	2.5 ± 0.4

Numbers represent the mean of three determinations ± SEM.

### Behavioural analyses

#### Morris water maze

**Fig. 1:**
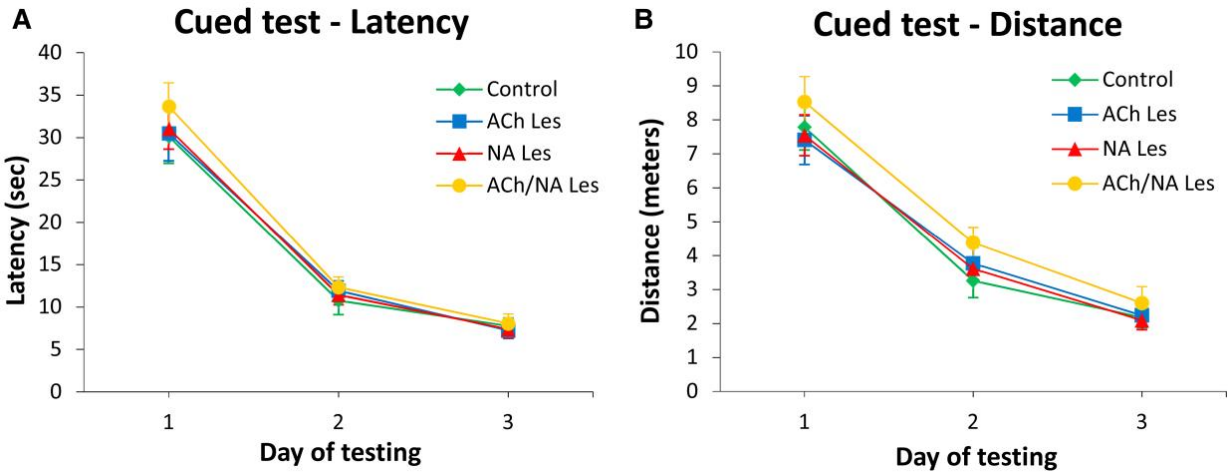
**Performance in the cued version of the Morris water maze test.** In this task, the visually cued platform was moved to a new quadrant on each of the four daily trials, and average escape latencies (**A**) and swim distances (**B**) were recorded. In this test, the animals (*n* = 15 in each group) did not differ from each other (two-way mixed ANOVA main group effect on latency, *F*(3,56) = 0.35; on distance, *F*(3,56) = 1.01; all *P* > 0.05, n.s.). Each point represents the mean value ± SEM for the block of four trials administered each day, over the three training days

When tested in the cued version of the water maze task at about 16 weeks post-lesion (Fig. 1A, B), the animals in all groups improved their performance over time (two-way mixed ANOVA, effect of day on latency, *F*(2,112) = 225.29; on distance, *F*(2,112) = 171.20; both *P* < 0.001) and did not differ from each other (main group effect on latency, *F*(3,56) = 0.35; on distance, *F*(3,56) = 1.01; group × day on latency, *F*(6,112) = 0.22; on distance, *F*(6,112) = 0.28; all n.s.). This test used a visible and signalled platform moved to a different position on each of the four daily trials, and was adopted, along with the grid and bridge tests to rule out non-specific, lesion-induced, sensory-motor impairments that would affect search navigation in the pool.

**Fig. 2:**
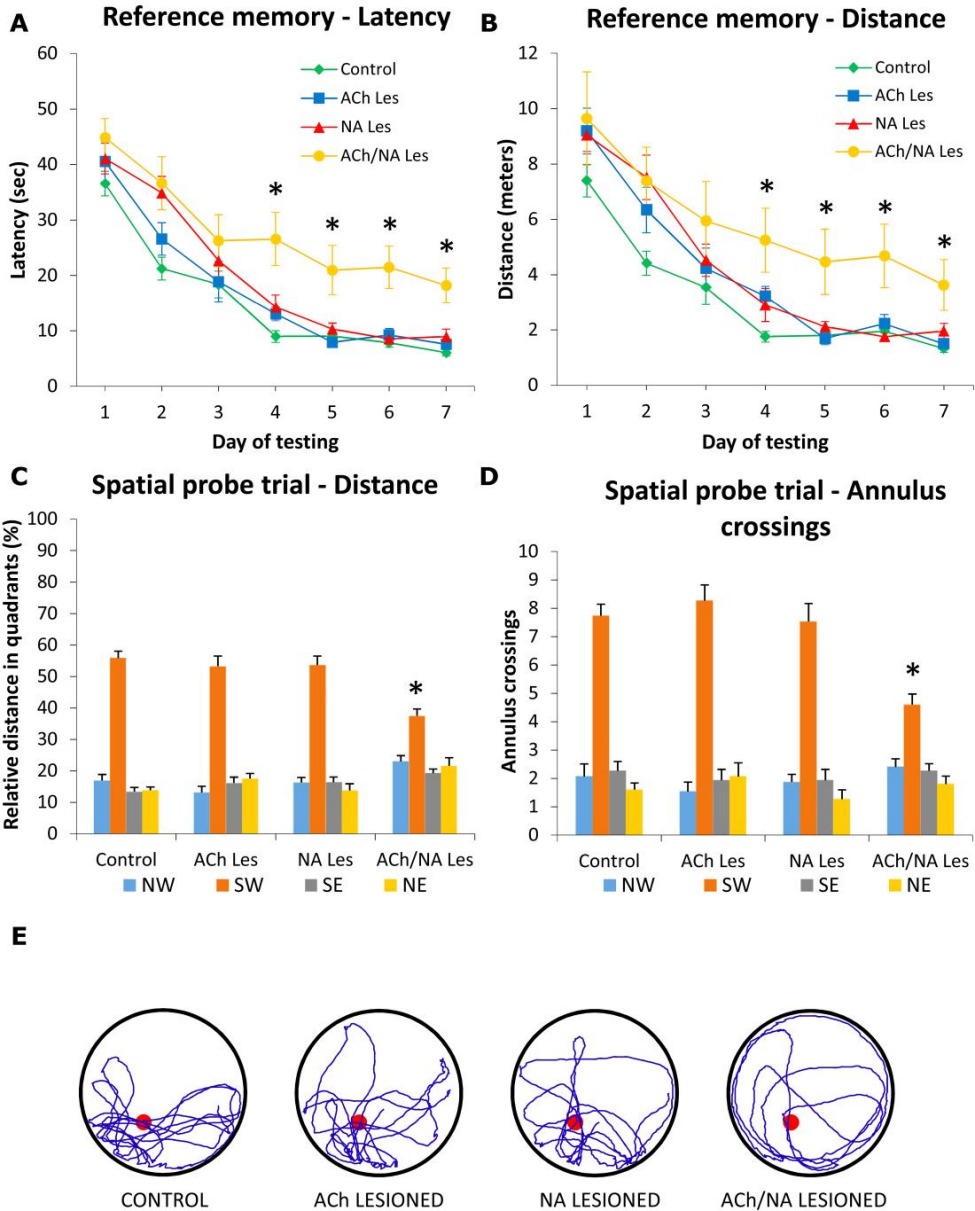
**Morris water maze, reference memory test at about 17 weeks post-lesioning.** Average escape latencies (**A**) and swim distances (**B**) required to the animals in the various groups (*n* = 15 in each group) to locate the submerged platform during the acquisition of the spatial navigation task. Each point represents the mean value for the block of four trials on each of the seven consecutive days of testing ± SEM (two-way mixed ANOVA, main group effect on latency, *F*(3,56) = 6.3; on distance, *F*(3,56) = 2.9, both *P* < 0.05; effect of day on latency, *F*(6,336) = 146.2; on distance, *F*(6,336) = 127.9; in both cases *P* < 0.001; group × day interaction for latency, *F*(18,336) = 1.8; for distance, *F*(18,336) = 1.8; both *P* < 0.05, followed by Tukey *post hoc* test). Lower diagrams illustrate the mean relative distance swum (**C**) and the average number of annulus crossings (**D**) in each quadrant during the spatial probe trial, upon removal of the escape platform. In (**E**), the actual swim paths taken by representative rats from the different groups are illustrated. Similar to controls, animals in the single ACh or NA lesion groups exhibited equally efficient performances and a pronounced bias for the original platform site in the training (SW) quadrant. By contrast, the ACh/NA double-lesioned animals appeared severely impaired on this task (two-way mixed ANOVA, effect of quadrant, *F*(3,168) = 198.5; group × quadrant interaction, *F*(9,168) = 6.4; both *P* < 0.001; effect of annulus position, *F*(3,168) = 190.6; group × annulus interaction, *F*(9,168) = 6.3; both *P* < 0.001, followed by Tukey *post hoc* test). Asterisks indicate significant difference from control, ACh lesion and NA lesion groups at *P* < 0.05.

Group performances in the place learning test are shown in Fig. 2A, B. All animals initially required about 37–45 s and 7–10 m to locate the submerged platform and improved significantly with repeated training (two-way mixed ANOVA, effect of day on latency, *F*(6,336) = 146.2; on distance, *F*(6,336) = 127.9; in both cases *P* < 0.001). However, whereas animals in the control, ACh lesion and NA lesion groups learned rapidly to locate the platform (so as to require 6–9 s and 1–2 m on the last day) and did not differ from each other, the double-lesioned animals performed much less efficiently throughout the testing period. Two-way mixed ANOVA revealed a significant effect of group (*F*(3,56) = 6.3; *F*(3,56) = 2.9 for latency and distance, respectively, both *P* < 0.05), as well as a group × day interaction (for latency, *F*(18,336) = 1.8; for distance, *F*(18,336) = 1.8; both *P* < 0.05), this latter reflecting the poor performance of double-lesioned subjects, compared with animals in the control, ACh lesion and NA lesion groups, particularly during the last 4 days of testing (Tukey *post hoc* test, *P* < 0.05 for both latency and distance). Swim speed, monitored during the execution of the navigation task to provide an additional measure of motor ability, did not differ between groups (*F*(3,56) = 0.6; *P* > 0.6; n.s.), and averaged 0.2–0.3 m/s across all testing days.

Fig. 2C, D illustrates animals’ ability to locate the platform site during the spatial probe trial on day 7. In general, the animals in the control, ACh lesion and NA lesion groups exhibited a pronounced bias for the original platform site and swam primarily in the training (SW) quadrant. By contrast, such spatially focused search behaviour appeared significantly less efficient in double-lesioned animals, that tended to distribute their swimming equally in all quadrants. Two-way mixed ANOVA on distance revealed a significant effect of quadrant (*F*(3,168) = 198.5; *P* < 0.001), as well as a group × quadrant interaction (*F*(9,168) = 6.4; *P* < 0.001). Subsequent analyses (one-way ANOVA + Tukey *post hoc* test), as well as inspection of actual swim paths (Fig. 2E), confirmed that the double-lesioned animals swam significantly less in the training quadrant than those in the other groups (*F*(3,56) = 10.0; *P* < 0.001; Fig. 2C). This observation was further confirmed when analysing the number of crossings over the annuli (Fig. 2D). In fact, there was a significant effect of annulus position (*F*(3,168) = 190.6; *P* < 0.001) and a group × annulus interaction (*F*(9,168) = 6.3; *P* < 0.001), indicating that the animals distributed their swim differently. Again, a clear-cut bias for the original platform site was exhibited by the animals in the control, ACh lesion and NA lesion groups, which crossed over the platform annulus significantly more than the double-lesioned, animals (*F*(3,56) = 10.7; *P* < 0.001; Tukey *post hoc* test *P* < 0.05), and did not differ from each other. Notably, the total number of annulus crossings did not differ among groups (*F*(3,56) = 2.1; *P* > 0.1; n.s.), suggesting an active, albeit not equally effective, search behaviour in all animals.

**Fig. 3:**
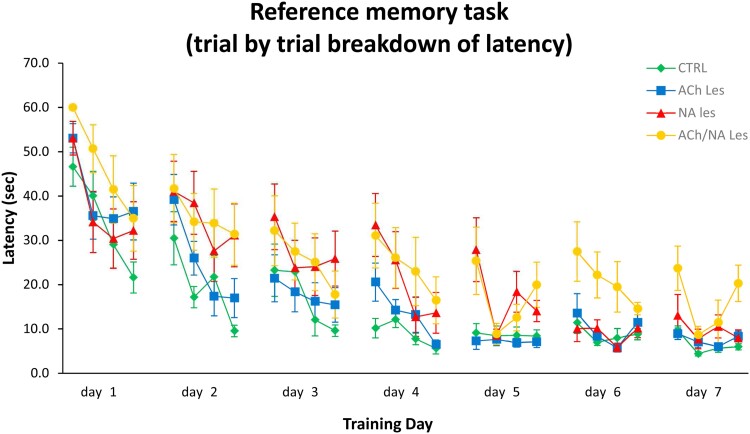
**Reference memory performance in the MWM test.** A trial-by-trial breakdown of the latency data is illustrated for the four experimental groups (*n* = 15 in each group). Initially, all animals behaved similarly and decreased their latency to find the platform. From day 4 onwards, animals in the control and single ACh lesion groups showed a steady performance both within and between the training days, a pattern that those in the single NA lesion group exhibited only in the last two testing days. By contrast, although capable of improving the latency to find the platform within one training day, the animals in the ACh/NA double lesion group did not seem to be able to retain this information to the next training day (three-way ANOVA, effect of groups, *F*(3,1568) = 40.7; *P* = 0.0001; effect of trial, *F*(3,1568) = 33.2; *P* = 0.0001; effect of day, *F*(6,1568) = 101.5; *P* = 0.0001; trial × day, *F*(18,1568) = 2.3; *P* = 0.0019; no significant group × day, group × trial or group × day × trial interactions).

To investigate the exact nature of the deficits exhibited by the double-lesioned animals, as opposed to the other groups, the reference memory data were analysed on a trial-by-trial basis (Fig. 3). On the first three testing days, the latency to find the platform was fairly similar in the four groups. All animals required a relatively longer latency on the first trial, but improved their performance on the subsequent trials across days (three-way ANOVA, effect of trial, *F*(3,1568) = 33.2; *P* = 0.0001; effect of day, *F*(6,1568) = 101.5; *P* = 0.0001; trial × day *F*(18,1568) = 2.3; *P* = 0.0019). Starting from day 4, the control and the ACh-lesioned animals reached a steady performance, with similar latency values between the last and the first trial of two consecutive days. By contrast, the NA-lesioned and the double-lesioned subjects exhibited latencies in the first trial that were higher than those recorded in the last trial of the previous day, in spite of a general improvement seen across trials within a single training day. Interestingly, however, the NA-lesioned animals reached a control-like performance on the last two testing days, whereas the impaired pattern exhibited by the double-lesioned animals remained unchanged up to day 7 (three-way ANOVA, effect of groups, *F*(3,1568) = 40.7; *P* = 0.0001; no significant group × day, group × trial or group × day × trial interactions).

**Fig. 4:**
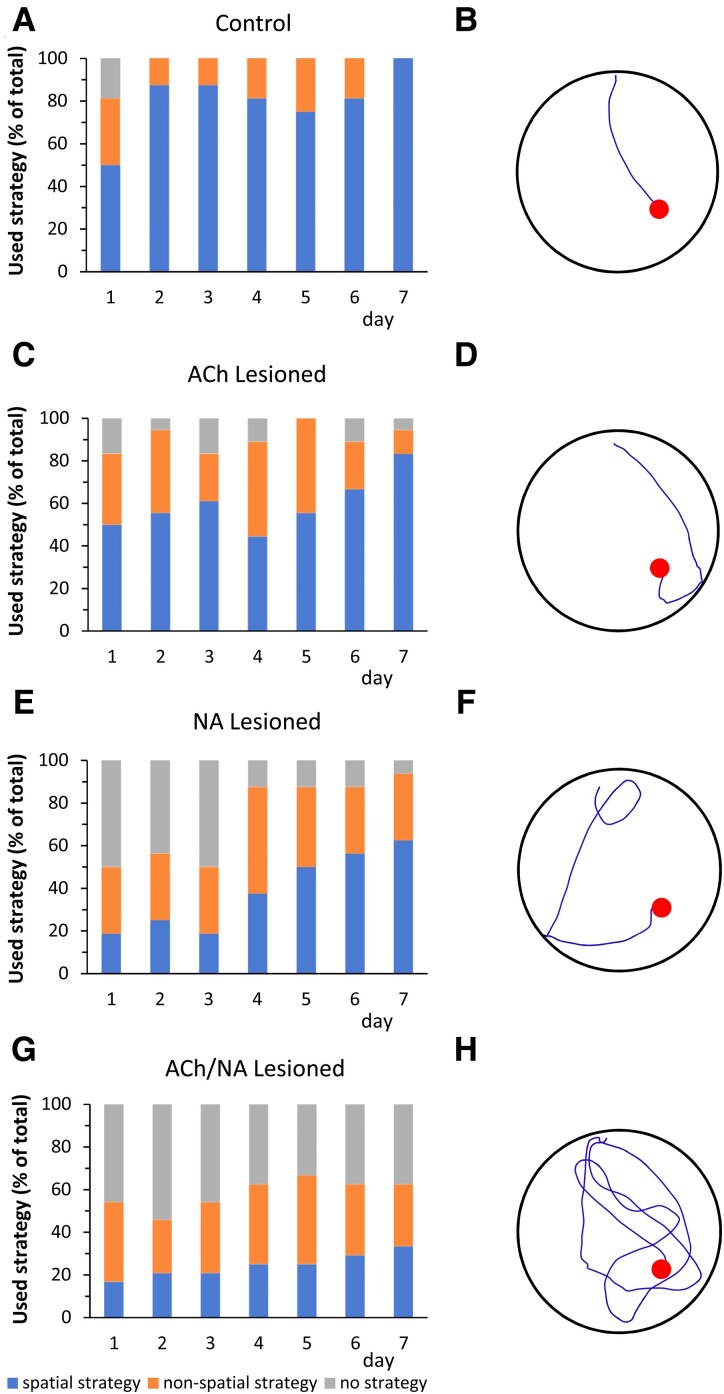
**Search strategy used during the fourth trial on each day of the MWM test.** The percentages of animals from the four groups (control, ACh-lesioned, NA-lesioned and ACh/NA double-lesioned animals, *n* = 15 in each group) using either spatial, non-spatial or no search strategy are presented as bar graphs (**A–D**). Sample swim paths from representative animals in the respective groups are illustrated as well (**E–H**). Starting from the second testing day, almost all animals in the control group adopted a spatial search strategy (**A**), and were able to locate the platform quickly (**E**). A similar behaviour was exhibited also by approx. 75% of the animals in the single ACh-lesioned group, particularly in the last two testing days (**B, F**). The single NA-lesioned animals differed from the controls in that they performed rather poorly in the first three testing days (i.e. about 50% of the animals showed no strategy), but improved dramatically with repeated trials, so that about 90% of them clearly used a search strategy in the last four testing days (**C, G**). About 42% of the animals in the ACh/NA double lesion group, on the other hand, were not able to use a spatial or non-spatial search strategy (**D, H**), and remained significantly impaired throughout the seven testing days (Pearson chi-square test, effect of groups, Pearson χ^2^_(420, 6)_ = 93.7; *P* = 0.0001; effect of days, Pearson χ^2^_(420, 12)_ = 22.0; *P* = 0.0372).

Further analyses aimed at evaluating the search strategy adopted to locate the platform during the execution of the fourth trial of the reference memory task on each of the seven testing days, i.e. when the animals have supposedly acquired the task contingency and have reached the best performance level for that particular day. Thus, the swim paths recorded from the subjects in the control, ACh lesion, NA lesion and ACh/NA double lesion groups were analysed by an observer blind to the animals’ identity and referred to one of the following categories: (1) use of spatial strategy; (2) use of non-spatial strategy and (3) no apparent strategy (see Materials and Methods). Swim paths recorded during the fourth trial on the seventh day from representative animals in the various groups were also selected (Fig. 4).

Control animals predominantly used a spatial strategy to locate the hidden platform, a pattern partly exhibited also by the animals in the ACh lesion group which, particularly on the last 2 days of testing, did not differ from controls (compare, e.g. A, E with B, F in Fig. 4). The majority of animals in the NA lesion and double lesion groups, by contrast, exhibited a poor spatial search during the first 3 days of testing, but they differed markedly in the strategy adopted in the subsequent 4 days. Thus, whereas a spatial strategy was progressively acquired by an increasing number of NA-lesioned animals, reaching on average 56% of the total number in the last three testing days (Fig. 4C, G), this was not the case for the double-lesioned animals, and only 29% of them used a spatial strategy, on average, in the last 3 days (Fig. 4D, H). Interestingly, in the control, ACh lesion and NA lesion groups, the progressive adoption of a spatial strategy by an increasing number of animals across days was associated with a concurrent reduction of the number of animals falling within the ‘no strategy’ category (respectively 0%, 5.6% and 10.4%, on average, in the last 3 days). By contrast, approximately 42% of the double-lesioned animals failed to develop a strategy, in spite of repeated training, and showed an overall poor performance across all testing days (effect of groups, Pearson χ^2^_(420, 6)_ = 93.7; *P* = 0.0001; effect of days, Pearson χ^2^_(420, 12)_ = 22.0; *P* = 0.0372).

#### Radial arm water maze

**Fig. 5:**
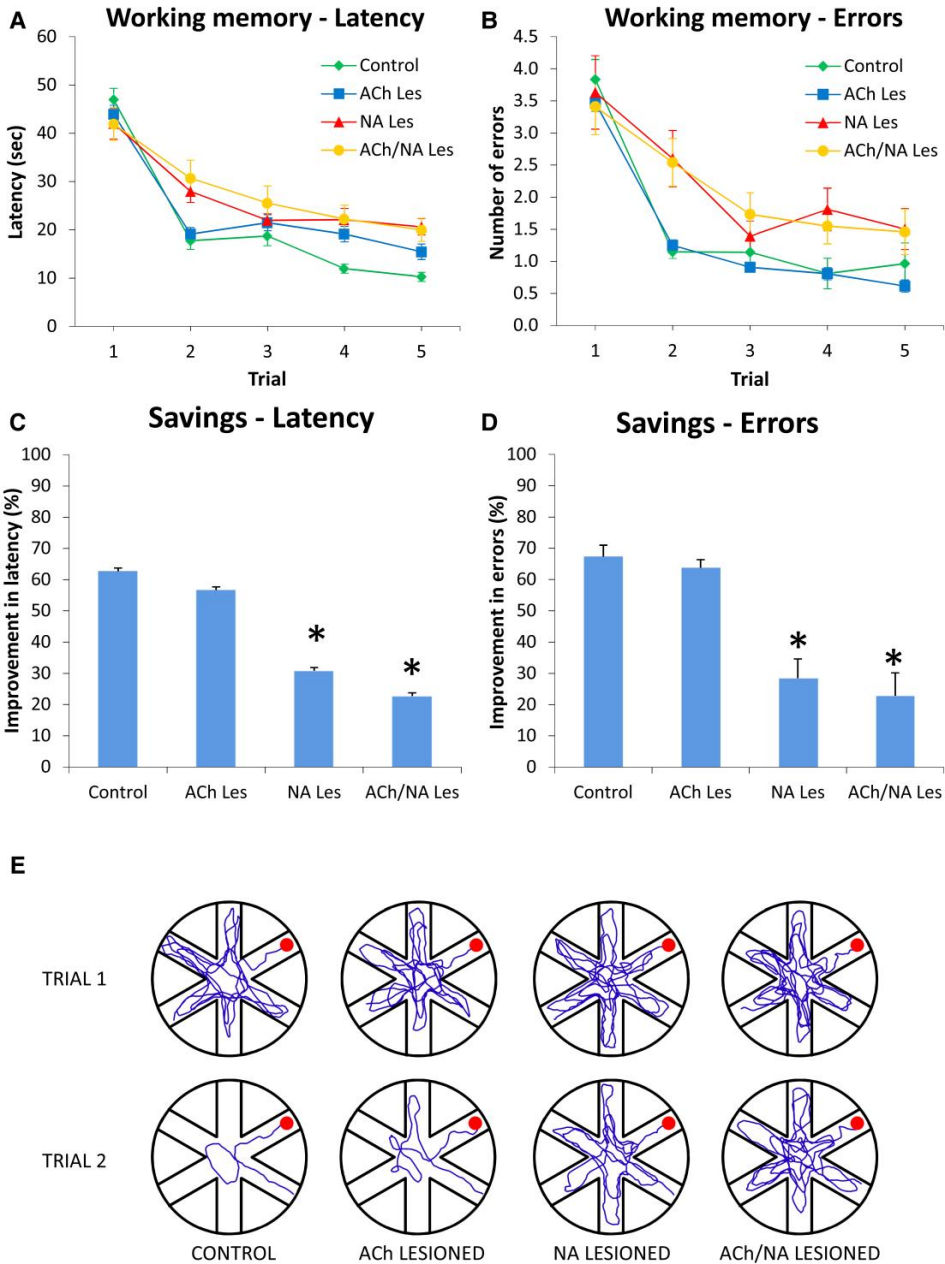
**Radial arm water maze, working memory performance at about 19 weeks post-lesioning.** Latency (**A**) and number of entry errors (**B**) required by the four groups (control, ACh-lesioned, NA-lesioned and ACh/NA double-lesioned animals, *n* = 15 in each group) to find the hidden platform in the radial arm water maze task. Each sample point represents the mean latency and errors recorded during each 60 second-trial over 5 consecutive testing days ± SEM (two-way mixed ANOVA, main group effect on latency, *F*(3,56) = 3.29; on errors, *F*(3,56) = 2.89; both *P* < 0.05; effect of trial on latency, *F*(4,224) = 164.21; on errors, *F*(4,224) = 92.75, both *P* < 0.001; group × trial interaction for latency, *F*(12,224) = 4.49; for errors, *F*(12,224) = 2.57, both *P* < 0.01). In the lower diagrams (**C** and **D**), performances are plotted as percent savings between trials 1 and 2 for latency and errors, respectively. In (**E**), the actual swim paths taken by representative animals from the different groups are illustrated. Note the rapid improvements in control and single ACh-lesioned animals and the marked impairments exhibited by animals in the single NA- and ACh/NA double-lesioned groups (one-way ANOVA for latency, *F*(3,56) = 13.27; for errors, *F*(3,56) = 19.17, both *P* < 0.001, followed by Tukey *post hoc* test). The asterisks in **C** and **D** indicate significant differences from the control and single ACh lesion groups at *P* < 0.01.

Group performances in the RAWM task are shown in Fig. 5A, B. In this task, the platform was moved to a new location daily, and the animals had to re-learn its position within the four trials of each testing day.

In general, animals in the control, ACh lesion, NA lesion and double lesion groups required longer latencies (on average 47 ± 2.3 s; 43.9 ± 1.9 s; 42 ± 3.2 s and 41.9 ± 3.4 s, respectively) and made more arm selection errors (i.e. entering an arm not containing the platform or an already visited one) during the first trial of each day. They then progressively reduced latency and errors (two-way mixed ANOVA, effect of trial on latency, *F*(4,224) = 164.21; on errors, *F*(4,224) = 92.75, both *P* < 0.001), but with a varying pattern of improvement (group × trial interaction, respectively *F*(12,224) = 4.49 and *F*(12,224) = 2.57 for latency and errors, both *P* < 0.01) suggesting differential abilities by the animals in the groups to ameliorate their performance across the trials. In fact, the closer inspection of data revealed that the control animals rapidly learned to locate the platform and markedly improved between the first and the second trials, a pattern exhibited also by animals in the ACh lesion group. By contrast, animals in the NA lesion group and the double-lesioned rats did not exhibit any such improvements (main group effect on latency, *F*(3,56) = 3.29; on errors, *F*(3,56) = 2.89; both *P* < 0.05; *post hoc* comparison with control and ACh lesion groups for both measures, *P* < 0.05).

In order to provide an additional measure of the learning efficiency in this task, the percentage improvement between trials 1 and 2 were analysed and plotted in terms of savings (Fig. 5C, D). Under these conditions, the animals in the control and ACh lesion groups reduced their latency and entry errors by about 63–57% and 67–64%, respectively, and did not differ from each other. By contrast, the percent improvement exhibited by the NA-lesioned and double-lesioned rats on both measures was significantly lower than that in control or ACh-lesioned animals (one-way ANOVA, savings for latency, *F*(3,56) = 13.27; for errors, *F*(3,56) = 19.17, both *P* < 0.001 followed by Tukey *post hoc* test, at *P* < 0.01) and did not exceed 30 and 23%, respectively (see also actual swim paths, in Fig. 5E).

## Discussion

The aim of the present study was to examine the relative contribution of the ascending cholinergic and noradrenergic projections, arising in the BF and LC regions, respectively, in the regulation of aspects of spatial learning and memory, using a well-established swim maze task. The study also sought to investigate whether an interaction between these two modulatory neurotransmitter systems would be important for maintaining a normal spatial memory performance in the rat. We found that selectively ablating cholinergic neurons in the BF of immature rats did not produce any clear-cut impairments in any of the water maze tasks used here to assess reference and working memory function.^37,48^ Conversely, and in keeping with previous observations in both immature^49^ and adult rats,^50^ selective disruption of noradrenergic LC neurons by the anti-DBH-saporin immunotoxin induced severe working memory deficits in the RAWM task, whereas reference memory abilities were seemingly unaffected. As an important and novel finding, a combined lesion of the cholinergic neurons in the BF and the noradrenergic neurons in the LC was sufficient to severely impair animals’ performance in the reference memory task and worsen the working memory deficits already evident following the single NA lesion.

### Effects of cholinergic depletion

In line with previous findings,^37^ the bilateral intraventricular injection of 0.4 μg IgG192-sap to neonatal rats (PD4) did not induce any non-cognitive (i.e. sensory-motor) impairments, as seen in the cued test. Moreover, in spite of a dramatic (≈80%) cholinergic neuronal and terminal fibre loss, the animals treated with 192 IgG-sap only performed as efficiently as controls in both the reference and working memory versions of the MWM task.

The lack of any reference or working memory deficits in the animals treated with 192 IgG-saporin alone argues that cholinergic innervation of the neocortex and hippocampus may not be primarily involved in the regulation of these cognitive domains^48,51,52^ but, rather, it would contribute to the maintenance of attentional processing, whose disruption may well affect task performance.^53,54^ However, the same lesion has been shown to induce reference memory impairments following intraventricular administration to adult rats.^40,55^ The varying efficacy of the immunotoxin when injected to neonatal or adult rats may reflect differences in the expression pattern of the low-affinity Nerve Growth Factor receptor (p75NTR), recognized and bound by the antibody moiety of the immunotoxin. In fact, the intensity of p75NTR immunoreactivity in basal forebrain cholinergic neurons reaches near-adult levels between the first and the second post-natal week,^56^ whereas its expression is relatively weak at PD4-PD8,^57^ i.e. the time when the lesions were administered in the present study. Alternatively, but not necessarily in contrast, it is possible that the deficits observed following an adult lesion may be associated with the concurrent disruption of cerebellar Purkinje cells,^42,58^ known to participate in the processing of spatial learning and memory information.^59-61^ The demonstration of a functional interplay between cerebellar circuitry and ascending regulatory transmitter systems is an interesting hypothesis *per se*, and future studies entailing discrete cerebellar injections of the 192 IgG-saporin immunotoxin, with or without simultaneous lesioning of the BF cholinergic neurons, will be necessary to address it further.

A third possibility is that other neurotransmitter systems may functionally compensate for the loss of cholinergic afferents and, in fact, marked increases in cortical and hippocampal NA tissue levels have previously been reported following neonatal 192 IgG-saporin lesions.^37,48^ In the hippocampus, these compensatory neurochemical responses may occur in afferent DBH + fibres from either the LC or the superior cervical ganglia (SCG). As to the former, they are likely to reflect increased transmitter turnover in resident (rather than outgrowth of novel), LC-derived NA fibres, as demonstrated by the similar density in hippocampal noradrenergic innervation exhibited by ACh-lesioned and control animals (Supplementary Table 1). Sprouting of SCG-derived sympathetic fibres in the hippocampus of animals with neonatal septal cholinergic lesions,^62,63^ results from accumulation of locally produced Nerve Growth Factor (NGF), no longer internalized by the degenerating cholinergic fibre terminals,^64,65^ and it is virtually identical to what has been observed here (see Supplementary material). Although previously reported to locally promote restorative events,^66,67^ this massive sprouting response, as observed here, does not appear to compensate for the loss of cholinergic and/or noradrenergic afferents in the hippocampus, nor does it seem to contribute to the behavioural sparing exhibited by ACh-lesioned animals.^53^ In fact, no clear-cut impairments can be detected in animals with a similar cholinergic lesion even after the sympathetic sprouting was prevented by surgically removing both SCG (unpublished observations).

### Effects of noradrenergic depletion

As discussed previously,^38^ the high efficiency of the αDBH-saporin toxin to ablate LC noradrenergic neurons, as opposed to the somewhat milder effects of the 192 IgG-saporin on BF cholinergic neurons, points to ontogenic differences in the expression pattern of the respective surface markers onto which the antibody moiety of toxin conjugate should bind to allow internalization of the toxic lectin. In fact, the expression of DBH is already ongoing prenatally^68^ and reaches an adult-like pattern immediately after birth.^69-71^ It is therefore plausible that due to higher numbers of DBH-presenting neurons or more dense DBH expression onto the same neurons, or both, the toxin can get easier access to the cell and act more efficiently.

In contrast with the above observations, no sympathetic sprouting was detected in single NA-lesioned animals (Supplementary material). This is not surprising, since LC-derived noradrenergic fibres innervating the hippocampus (unlike ingrowing sympathetic fibres) do not normally express the p75NTR and they are not under trophic regulation by locally produced NGF.^48,72^ Thus, in no case can any behavioural sparing in these animals be attributed to sympathetic sprouting. All animals injected with αDBH-saporin showed severe and consistent impairments only in the working memory task. These data appear to confirm and extend previous findings of NA-related effects on spatial navigation induced by aging,^73,74^ pharmacological manipulations,^75,76^ non-selective neurotoxin^77,78^ or—more recently—selective immunotoxin lesions.^49,50,79^ Notably, the working memory deficits detected here in animals with neonatal intraventricular administration of αDBH-saporin (see also Pintus *et al*.^49^) are very similar in magnitude to those reported previously following discrete intrahippocampal injections of the same immunotoxin.^79^ This is of importance, as it would strongly suggest that—at least in the rat and with the experimental paradigm adopted here—the NA-mediated events critical for the normal execution of a working memory task may take place also at the level of the hippocampus. Moreover, the time course of those investigations (≈8–12 months) supports the notion of a high stability over time of the anatomical and functional effects induced the neonatal lesioning treatment.

On the other hand, the lack of any significant reference memory impairments, as reported here in animals with a single NA lesion, is somewhat puzzling also in view of previous studies where such deficits were indeed detected following non-selective lesioning^30,32,80,81^ or reversible LC inactivation.^75^ Based on the notion of a noradrenergic involvement in multiple cognitive domains,^22,82^ it is reasonable to assume a role for NA in more demanding aspects of cognition, namely those related to working memory, where information need to be transiently stored and rapidly retrieved and processed to drive goal-directed behaviour.^50^ Alternatively, the lesion-induced NA loss may have induced subtle reference memory deficits difficult to detect with the testing paradigm adopted here and rather modest, at best.^83^ The trial-by-trial and swim path analyses revealing initial deficits normalized by the fifth training day, suggest that this may be the case.

### Effects of combined cholinergic and noradrenergic depletion

We found here that simultaneous loss of NA and ACh inputs, by combined lesions of noradrenergic neurons in the LC and cholinergic neurons in the BF, respectively, induced significant deficits in reference memory and exacerbated the impairments in working memory performance caused by the single NA lesion alone.

The reference memory deficits exhibited by the double-lesioned animals, as opposed to the apparent lack of impairments in either single-lesioned groups suggests that the two regulatory transmitter systems positively interact to sustain a normal performance in the reference memory task. Thus, a cholinergic–noradrenergic interplay is probably required for regulating certain aspects of spatial learning and memory (i.e. those related to the less demanding reference memory abilities) whose alterations may not be immediately evident when only one of the two trasmitter systems is dysfunctional. If so, it is possible that with one of the two systems down, as obtained here with the production of single lesions, the functions normally regulated by that system would exhibit only marginal or no alterations, and be readily reinstated to a near-normal status, possibly thanks to the compensatory activity of the intact one^37^ and/or the adoption of alternative search strategies, such as those based on heading vectors.^84^ When, on the other hand, both systems are severely depleted, the resulting lack of afferent regulatory control leads to the manifestation—or exacerbation—of the deficits in one or more aspects of cognitive performance, with a much reduced capacity to devise and use any strategy. The relative high rate of double-lesioned animals that were found falling within the ‘no strategy’ category in the swim path analysis (≈42%, see above) suggests that this may be the case. These events would be even more evident when the ablation of the relevant neuronal systems is highly selective (as it is here) and thus not hampered by concurrent damage to other structures. The finding of reference memory deficits in the double-lesioned animals in this study appears in keeping with the observations of Grigoryan *et al*.^85^ following excitotoxin and neurotoxin injections in the BF nuclei and the dorsal noradrenergic bundle, respectively, but it is also at variance with them, as no significant or very mild impairments were detected in this task in animals with single ACh or NA lesions. Likewise, in the present study, only NA single- and ACh–NA double-lesioned animals did actually exhibit working memory deficits, a series of discrepancies likely to depend upon the different selectivity of the lesioning treatments in the two investigations.

Finally, the fact that double-lesioned animals exhibited significant impairments in both reference and working memory, despite the marked presence of SCG-derived sympathetic fibres in the hippocampus appears to confirm earlier reports that these ectopically outgrowing fibres may indeed not be functional.^53,86,87^

Several considerations can be drawn from these findings: (i) The functional convergence of the two regulatory transmitter systems (i.e. the BF-derived cholinergic and the LC-derived noradrenergic) for the regulation of spatial learning and memory is likely to occur in the hippocampus. (ii) As seen above for reference memory, the two systems are likely to interact at hippocampal level also to regulate in particular the more demanding working memory function, possibly via their concurrent involvement in the attention/arousal domains. (iii) The somewhat higher magnitude of the effects induced by the double lesion, as compared with those induced by the NA lesion alone seems to highlight the relatively modest cholinergic contribution to the effects, particularly those related to working memory which, thus, appears to more crucially be under noradrenergic control. (iv) Ectopic, SCG-derived fibre ingrowth does occur in the denervated hippocampus as a result of the neonatal cholinergic lesion, but it is unlikely to contribute to any observed behavioural sparing.

To the best of our knowledge, this is the first study where the anatomical and cognitive effects of single versus concurrent cholinergic and noradrenergic depletion were investigated using highly selective immunotoxins with a very accurate counterbalanced and delayed administration design. A number of previous studies have, in fact, addressed the same issue by adopting far less specific lesioning procedures and/or pharmacological manipulations, often with conflicting results.^28,30,32,33,53,77,85,88-90^

Clearly, the production of massive cholinergic–noradrenergic depletions in developing animals, as done here, does not seem to best mimic the progressive nature of neuronal degeneration, as seen in AD patients. In particular, the long-term effects of these combined lesions remain to be investigated, also in light of their observed stability (over at least a 8–12 months period) when administered individually.^37,38^ Thus, further longitudinal studies, with longer survival times and analyses at different time-points post-surgery, will be necessary to more adequately address the development and time course of the lesion-induced cognitive impairments, as well as possible additive/synergistic effects.

Interestingly, however, we have previously shown that reference or working memory functions, severely perturbed even many months following selective cholinergic or noradrenergic lesions, can be ameliorated/normalized by transplanted cholinergic^55,91^ or noradrenergic^49,79^ neural precursors promoting reinnervation of the depleted hippocampal target regions. These restorative actions apply as well to the lesion-induced pathological transformation of resident peptides or proteins,^49,55^ but presently there are no data substantiating similar effects on tissue pathology possibly triggered by a concurrent cholinergic–noradrenergic loss.

Future studies will investigate the long-term consequences of these dual lesions and also address whether locally restoring both cholinergic and noradrenergic neurotransmission is sufficient to normalize reference/working memory abilities and reinstate physiological protein expression in animals with double lesions.

## Conclusion

Current hypotheses on the possible causes of AD, posit that both the cholinergic and noradrenergic transmitter systems contribute to the onset and progression of memory dysfunction. The novel double lesion paradigm used in this study suggests that these systems normally regulate distinct cognitive domains but they also interact in some aspects of spatial learning and memory. The present findings may thus help to address their potential role as targets for future therapeutic interventions.

## Supplementary Material

fcac338_Supplementary_Data

## Abbreviations

Aß =: beta amyloid peptide
ACh =: acetylcholine
AChE =: acetylcholinesterase
AD =: Alzheimer’s disease
BF =: basal forebrain
ChAT =: choline acetyltransferase
DBH =: dopamine beta hydroxylase
HSD =: honestly significant difference
LC/SubC =: locus coeruleus/subcoeruleus complex
MWM =: Morris water maze
NA =: noradrenaline
NBM =: nucleus basalis magnocellularis
NFTs =: neurofibrillary tangles
NGF =: nerve growth factor
PD =: post-natal day
RAWM =: radial arm water maze
SCG =: superior cervical ganglia
Sept/vDBB =: septum/vertical limb of the diagonal band of Broca
SW =: southwest
