# Supplementary material for: Acetylcholine and noradrenaline differentially regulate hippocampus-dependent spatial learning and memory
Source: Brain Commun. 2022 Dec 22;5(1):fcac338. doi: 10.1093/braincomms/fcac338 (PMC9825812; doi:10.1093/braincomms/fcac338)
Supplement: fcac338_Supplementary_Data [file fcac338_supplementary_data.pdf]

Supplementary material

ACETYLCHOLINE AND NORADRENALINE DIFFERENTIALLY REGULATE HIPPOCAMPUS-DEPENDENT SPATIAL LEARNING AND MEMORY

Gioacchino de Leo, Rosario Gulino, Marino Coradazzi, Giampiero Leanza

Methods

Histological procedures

At the end of the experimental period, at about 20 weeks post-lesion, the rats were deeply anaesthetized (sodium pentobarbital, 60 mg kg-1 i.p.) and transcardially perfused with saline followed by ice-cold 4% phosphate-buffered paraformaldehyde (pH 7.4). The brains were rapidly dissected out, postfixed for 2 h in the same fixative and then kept at 4°C in a phosphate-buffered 20% sucrose solution until they had sunk. Using a freezing microtome, four series of coronal sections at 40 µm thickness were cut from the level of the medial septum/vertical limb of the diagonal band of Broca (Sept/vDBB) through the nucleus basalis magnocellularis (NBM), thus comprising the frontal cortex and hippocampus, and from the caudal mesencephalon to the medulla oblongata, thus comprising the LC/SubC complex.

Immunohistochemistry was conducted on free-floating sections following a standard avidin-biotin ABC procedure[1]. Endogenous peroxidase activity was first quenched by a 10 min incubation in 10% methanol with 3% hydrogen peroxide in 0.02 M potassium phosphate-buffered saline (KPBS, pH 7.4), followed by preincubation in 5% normal goat or horse serum (NGS or NHS, both from Immunological Sciences, Rome, Italy) and 0.3% Triton (Merck Darmstadt, Germany) in KPBS for 1 h. The sections were then exposed to the primary antiserum diluted in 2% NGS or NHS and 0.3% Triton in KPBS for 16-24 h, followed by 1 h incubation with 1:200 goat anti-rabbit or horse anti-

mouse biotinylated IgGs (Vector, Burlingame, USA). The sections were then reacted with 0.025% diaminobenzidine (Sigma) and 0.01% hydrogen peroxide in KPBS for 2-5 min. In order to ensure consistency during morphometric analyses (see below), tissue processing and stainings were carried out under identical conditions, using all relevant sections at a time. Controls for nonspecific labeling were also used where the primary antibody had been omitted. All steps were performed at room temperature.

The following primary antisera were used: mouse anti-DBH monoclonal antibody (1:2000, Merck Millipore, Darmstadt, Germany); rabbit anti-choline acetyltransferase (ChAT) polyclonal antibody (1:1000, Immunological Sciences, Rome, Italy).

In order to visualize the effects of the toxin treatment on the cholinergic innervation in the neocortex and hippocampus, one series of sections was processed free-floating for acetylcholinesterase (AChE) histochemistry.[2] Briefly, the sections were incubated in sodium citrate, copper sulphate, sodium acetate, potassium ferricyanide, acetylthiocholine iodide and ethopropazine (Sigma). The brown reaction product was then intensified with ammonium sulphide and silver nitrate.

Microscopic analysis and quantitative evaluation

All analyses were conducted on coded slides by observers unaware of the group's identity. The efficacy of the single or double lesioning treatment was stereologically estimated on immunostained sections using the optical fractionator principle.[3] Analyses were carried out on cholinergic (i.e. ChAT-immunoreactive) and/or noradrenergic (i.e. DBH-immunoreactive) neurons in the basal forebrain nuclei and in the pontine LC/SubC region, respectively. Immunoreactive neurons in the Septum/vDBB were counted bilaterally from the level of the genu of the corpus callosum, rostrally, to the crossing of the anterior commissure, caudally, defining the lateral edge of the vDBB at the medial border of the olfactory tubercle. Immunoreactive neurons in the NBM were counted from the level of the caudal septum to the tail of the caudate-putamen.[4] DBH-immunoreactive neurons in the locus coeruleus region were counted bilaterally from the level of the ventral portion of the

parabrachial nucleus, rostrally, to the genu of the facial nerve, caudally. The counting included the more scattered noradrenergic neurons in the subcoeruleus region, just ventral to the LC proper.[5,6]
The sampling system consisted of an Olympus BH2 microscope (fitted with an X-Y motorized stage and a microcator to measure distances in the Z axis) interfaced with a colour video camera (Sony) and a personal computer. The CAST GRID® software (Olympus Denmark A/S, Albertslund, Denmark) was used to delineate the selected regions at 4x magnification, as well as to generate unbiased counting frames which were moved randomly and systematically until the entire delineated area was sampled. Using a 100x oil objective, unambiguously positive cells were identified and counted after excluding guard volumes from both section surfaces, in order to prevent problems of lost caps.

Lesion-induced changes in the density of cholinergic and/or noradrenergic innervation in the neocortex and hippocampus, relative to normal patterns, were assessed by densitometry using the Image 1.61 NIH freeware.[7] Briefly, measuring fields of consistent size (0.5 mm in diameter) were selected bilaterally from 3 different sections in the frontoparietal cortex, as well as in the CA1 subfield of the dorsal hippocampus and the dentate gyrus.[4,8,] Background density, as determined in a structure normally lacking the AChE-or the DBH-positive staining (i.e. the corpus callosum), was subtracted from each measurement. Relative optical density levels in the analysed regions were expressed as arbitrary units, averaging the values measured from the three sections.

Results

Morphological analyses

The infusion of the 192 IgG-saporin or the anti-DBH-saporin immunotoxins into the lateral ventricles of immature rats produced, at about 20 weeks post-lesion, a fairly complete loss of ChAT-immunoreactive (i.e. cholinergic) neurons in the basal forebrain nuclei Septum/vDBB and NBM (Supplementary Fig. 1 A-H) or DBH-immunoreactive (i.e. noradrenergic) cells throughout the

brainstem LC/SubC complex, with no obvious side differences (Supplementary Fig. 2 A-D). Notably, cholinergic neurons in the basal forebrain of the animals with a single NA lesion were largely unaffected (compare, e.g. C,G with A,E in Supplementary Fig. 1), so were the noradrenergic neuronal populations in the LC/SubC nuclei of single ACh-lesioned animals (compare A with B in Supplementary Fig. 2). By contrast, the same regions were observed almost totally devoid of cholinergic and noradrenergic neurons in double-lesioned animals (panels D,H in Supplementary Fig. 1 and D in Supplementary Fig. 2). Thus, the counterbalanced, two-stage toxin injection design adopted here produced no obvious differences in the efficiency of the lesions.

Consistent with previous observations,[9,10] neither the single, nor the double lesioning procedures had any clear-cut effects upon parvalbumin-immunoreactive neurons in the medial septum, the tyrosine hydroxylase-positive dopaminergic neurons in the substantia nigra pars compacta/ventral tegmental area or serotonergic neurons in the raphe nuclei. Likewise, calbindin-immunoreactive Purkinje cells in the cerebellar cortex appeared largely unaffected by either treatments (data not shown). Stereological estimates (Supplementary Table 1) revealed that the cholinergic neuronal depletion in the Septum/vDBB and NBM averaged 82% and 77%, respectively, in the animals with single ACh or double ACh-NA lesion, compared to Control. By contrast, and in keeping with previous findings,[8,9] the lesion-induced loss of noradrenergic neurons in the LC/SubC was far more severe, and averaged about 95% in single NA- and ACh-NA double-lesioned rats compared to control. The observed neuronal depletions were associated to a widespread ~70-80% loss of AChE- (panels B and D in Supplementary Fig. 3) or DBH-positive fibers (panels C and D in Supplementary Fig. 4) bilaterally in the fronto-parietal cortex and in the various subfields of the hippocampal formation, i.e. major target regions for the ascending cholinergic and noradrenergic projections. Consistently, in these areas, no obvious changes in the density of cholinergic or noradrenergic innervation could be detected in animals with single NA or ACh lesion, respectively (e.g. C in Supplementary Fig. 3 and B in Supplementary Fig. 4) compared to Controls (panels A in both Supplementary Figs. 3 and 4**)**, whereas the dramatic cholinergic and noradrenergic denervation of neocortical/hippocampal

regions was concurrently evident in ACh-NA double-lesioned animals (panels D in Supplementary Figs. 3 and 4; see also Supplementary Table 1). Interestingly, in this latter group, as well as in animals with single ACh lesion, thick, strongly DBH-immunoreactive, varicosities were observed to bilaterally invade the hippocampal dentate gyrus and hilus, where they scattered rather coarsely, with no clear-cut distribution pattern (arrows in Supplementary Fig. 4 F,H). These fibers, likely to reflect peripheral sympathetic sprouting of noradrenergic fibers outgrowing from the superior cervical ganglion in response to the cholinergic denervation,[11-14] were easily distinguishable from the finely distributed LC-derived innervation, and were not detected in Control and single NA-lesioned animals (Supplementary Fig. 4 E,G). As assessed by densitometry, these coarse fibers did not contribute significantly to the density of the DBH-immunoreactive processes in this subregion of the hippocampus (Supplementary Table 1).

Supplementary Table 1: Stereological estimates of ChAT- and DBH-immunoreactive neurons in the basal forebrain nuclei and locus coeruleus, respectively, and densitometric evaluations of AChE- and DBH terminal fiber density in the neocortical and hippocampal terminal regions

Group	Control (n = 15)	ACh Lesion (n = 15)	NA Lesion (n = 15)	ACh-NA Lesion (n = 15)
ChAT-ir neurons in Sept-DBB	5832.7 ± 250.2	1039.7 ± 88.2*	5600.0 ± 253.3	1032.7 ± 101.7*
ChAT-ir neurons in NBM	2313.0 ± 172.4	599.0 ± 92.5*	2435.7 ± 161.8	549.8 ± 64.4*
DBH-ir neurons in LC/SubC	1880.0 ± 248.0	1710.0 ± 132.0	103.3 ± 7.1*	100.7 ± 14.3*
AChE(+) fibers in Fr-Par Cx	47.3 ± 2.6	12.7 ± 1.8*	52.3 ± 2.8	13.3 ± 1.2*
AChE(+) fibers in CA1	69.0 ± 4.2	10.7 ± 0.3*	68.7 ± 2.0	14.0 ± 1.2*
AChE(+) fibers in CA3	73.7 ± 4.3	13.0 ± 0.6*	67.0 ± 4.5	10.7 ± 0.9*
AChE(+) fibers in DG	56.3 ± 1.3	11.3 ± 2.9*	58.3 ± 3.7	10.3 ± 0.9*
DBH-ir fibers in Fr-Par Cx	83.7 ± 10.8	74.4 ± 2.2	20.5 ± 3.3*	20.9 ± 1.9*
DBH-ir fibers in CA1	64.2 ± 5.9	69.8 ± 2.9	19.0 ± 2.5*	16.3 ± 1.4*
DBH-ir fibers in CA3	63.0 ± 2.3	67.1 ± 1.1	21.2 ± 1.3*	18.6 ± 3.6*
DBH-ir fibers in DG	60.9 ± 4.1	58.8 ± 3.8	21.0 ± 1.0*	16.6 ± 1.3*

Values represent the estimated total number of ChAT- and DBH-immunoreactive neurons in the basal forebrain nuclei (sept/DBB and NBM) and LC respectively, and the standardized relative density scores (± SEM) of AChE and DBH-positive innervation in each of the target areas in the fronto-parietal cortex and the various subdivisions of the hippocampal formation. Asterisks indicate significant difference from Control ($p < 0.001$).

References

1. Leanza G, Cataudella T, Dimauro R, Monaco S, Stanzani S. Release properties and functional integration of noradrenergic-rich tissue grafted to the denervated spinal cord of the adult rat. *Eur J Neurosci*. 1999;11:1789-1799.
2. Hedreen JC, Bacon SJ, Price DL. A modified histochemical technique to visualize acetylcholinesterase-containing axons. *J Histochem Cytochem*. 1985;33:134-140.
3. West MJ, Slomianka L, Gundersen HJ. Unbiased stereological estimation of the total number of neurons in thesubdivisions of the rat hippocampus using the optical fractionator. *Anat Rec*. 1991;231:482-497.
4. Aztiria E, Cataudella T, Spampinato S, Leanza G. Septal grafts restore cognitive abilities and amyloid precursor protein metabolism. *Neurobiol Aging*. 2009;30:1614-1625.
5. Amaral DG, Sinnamon HM. The locus coeruleus: neurobiology of a central noradrenergic nucleus. *Prog Neurobiol*. 1977;9:147-196.
6. Grzanna R, Molliver ME. The locus coeruleus in the rat: an immunohistochemical delineation. *Neuroscience*. 1980;5:21-40.
7. Rasband WS, Bright DS. NIH Image: a public domain image processing program for Macintosh. *Microbeam Analysis Society Journal*. 1995;4:137-149.
8. Pintus R, Riggi M, Cannarozzo C, *et al*. Essential role of hippocampal noradrenaline in the regulation of spatial working memory and TDP-43 tissue pathology. *J Comp Neurol*. 2018;526:1131-1147.
9. Leanza G, Nilsson OG, Nikkhah G, Wiley RG, Björklund A. Effects of neonatal lesions of the basal forebrain cholinergic system by 192 immunoglobulin G-saporin: biochemical, behavioural and morphological characterization. *Neuroscience*. 1996;74:119-141.

10. Coradazzi M, Gulino R, Garozzo S, Leanza G. Selective lesion of the developing central noradrenergic system: short- and long-term effects and reinnervation by noradrenergic-rich tissue grafts. *J Neurochem*. 2010;114:761-771.

11. Crutcher KA. Sympathetic sprouting in the central nervous system: a model for studies of axonal growth in the mature mammalian brain. *Brain Res*. 1987;434:203-233.

12. Crutcher KA, Davis JN. Target regulation of sympathetic sprouting in the rat hippocampal formation. *Exp Neurol*. 1982;75:347-359.

13. Harrell LE, Parsons DS, Kolasa K. The effect of central cholinergic and noradrenergic denervation on hippocampal sympathetic ingrowth and apoptosis-like reactivity in the rat. *Brain Res*. 2005;1033:68-77.

14. Pappas BA, Davidson CM, Fortin T, *et al*. 192 IgG-saporin lesion of basal forebrain cholinergic neurons in neonatal rats. *Brain Res Dev Brain Res*. 1996;96:52-61.

Supplementary Figure legends

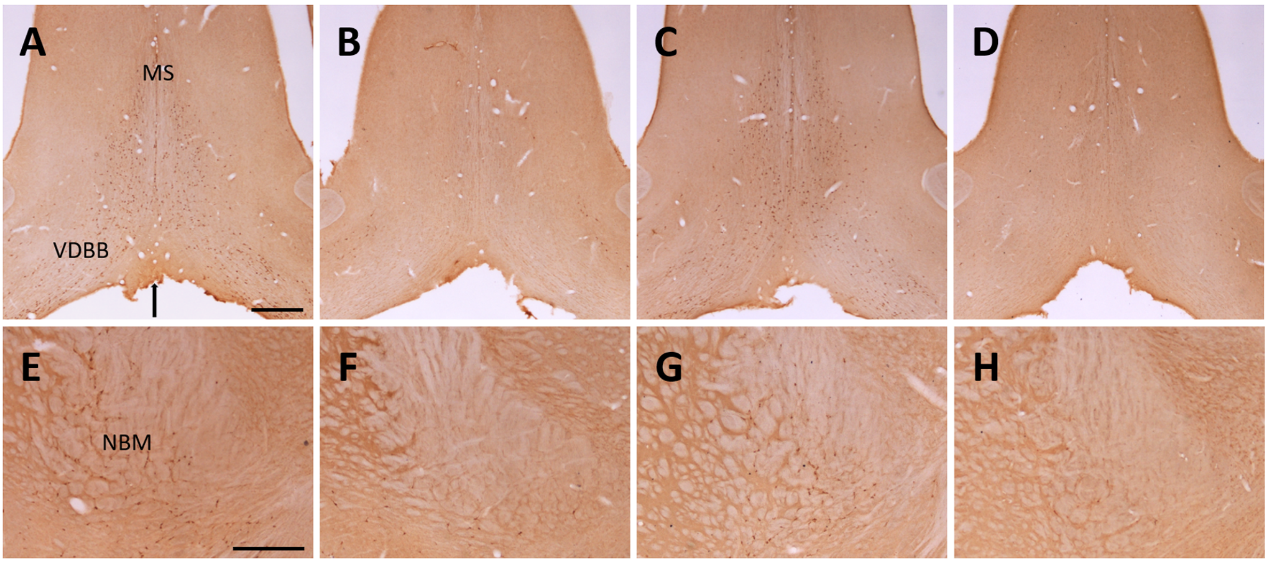

Supplementary Fig. 1 Representative examples of choline acetyltransferase (ChAT) immunostaining in coronal sections from the septum/vDBB (A–D) and NBM (E–H) in the various treatment groups. At about 20 weeks post-lesioning, marked depletions of ChAT immunoreactive neurons were evident in both the Single ACh-Lesioned (B, F) and the ACh/NA Double lesioned animals (D, H), whereas the same regions appeared unaffected in Single NA - Lesioned animals (C, G), compared to Controls (A, E). The arrow in (A) indicates the midline. Scale bars in (A) and (E): 500 µm.

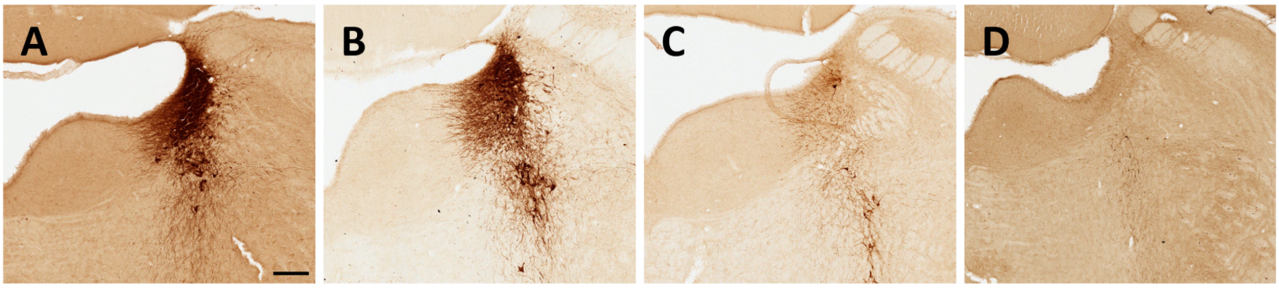

Supplementary Fig. 2 Representative examples of Dopamine-ß-Hydroxylase (DBH) immunostaining illustrating, on the coronal plane, the distribution of noradrenergic neurons in the LC/SubC regions of Control (A), Single ACh-Lesioned (B), Single NA-Lesioned (C) and ACh/NA Double Lesioned animals (D). Note in (C) and (D) the near complete loss of DBH immunoreactive neurons and proximal processes induced by the lesion, compared to the normal patterns in (A) and (B). Scale bar in (A): 250 µm.

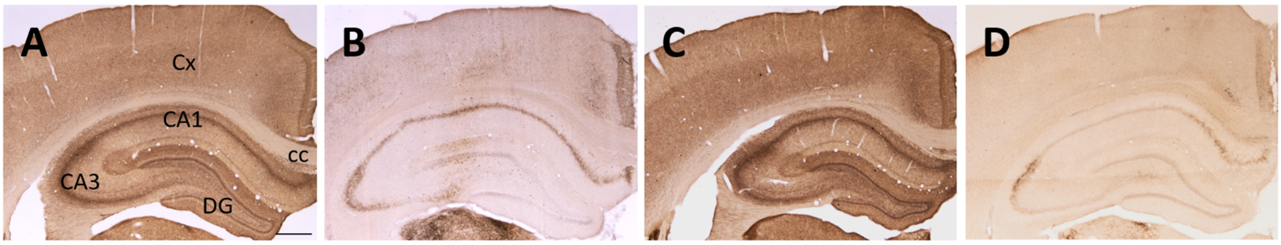

Supplementary Fig. 3 Representative examples of acetylcholinesterase (AChE) histochemistry showing, on the coronal plane, the extent of cholinergic denervation, in the various treatment groups, at about 20 weeks post-lesioning. Note the dramatic denervation induced by the toxin-conjugate in the neocortex and hippocampus of the Single ACh-Lesioned (B) and the ACh/NA Double lesioned animals (D) compared to the normal pattern exhibited by the Control (A) and the Single NA-Lesioned animals (C). Cx, fronto-parietal cortex; CA, cornu ammonis of the hippocampus; CC, corpus callosum; DG, dentate gyrus. Scale bar in (A): 500 µm;

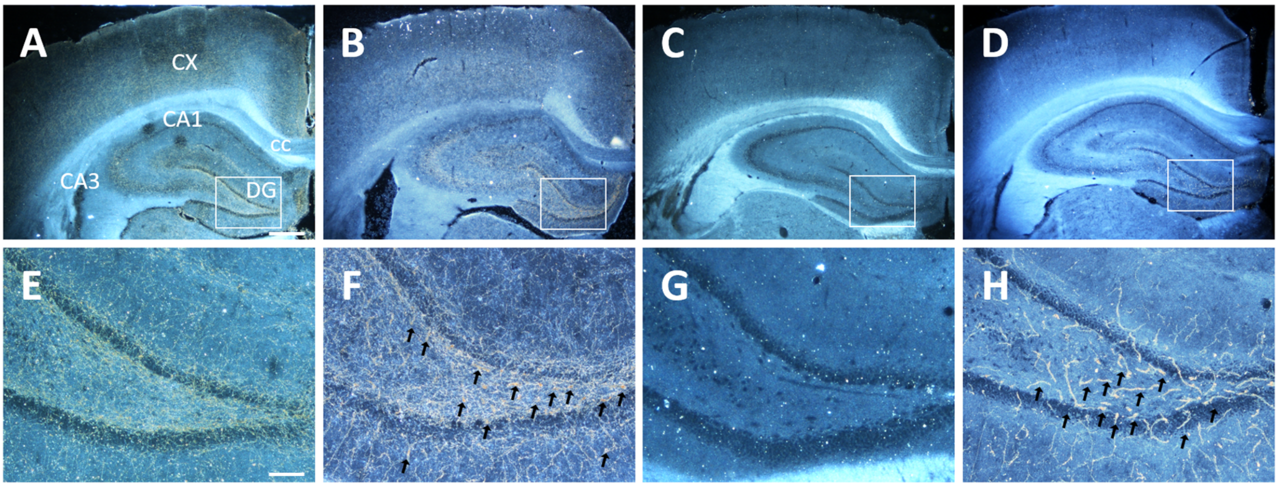

Supplementary Fig. 4 Representative examples of dark-field DBH-immunostaining illustrating the DBH-immunoreactive (i.e. noradrenergic) innervation in the fronto-parietal cortex and hippocampus from Control (A, E), Single ACh-lesioned (B, F), Single NA-lesioned (C, G) and ACh/NA Double-lesioned animals (D, H). Note the almost total loss of DBH-immunoreactive fibers in all target regions of Single NA-lesioned (C, G) and ACh/NA double-lesioned animals (D, H), compared to Control (A, E) and Single ACh-lesioned animals (B. F). Remarkably, in these latter and in the ACh/NA double-lesioned animals thick and coarse DBH-immunoreactive fibers, outgrowing from the superior cervical ganglia as a result of the early cholinergic denervation, were evident mainly in the DG (arrows in F and H) where they could easily be distinguished from the more fine and patterned DBH-immunoreactive fibers coming from the LC (compare F, H, with E). Cx, fronto-parietal cortex; CA, cornu ammonis of the hippocampus; CC, corpus callosum; DG, dentate gyrus. Photomicrographs in (E-H) are magnifications of the boxed areas in (A-D). Scale bar in (A) (for A-D): 500 µm; Scale bar in (E) (for E-H): 100 µm.
